# Host immune response to anti-cancer camptothecin conjugated cyclodextrin-based polymers

**DOI:** 10.1186/s12929-019-0583-0

**Published:** 2019-10-23

**Authors:** Yi-Fan Chen, Yen-Hsin Wang, Cing-Syuan Lei, Chun A. Changou, Mark E. Davis, Yun Yen

**Affiliations:** 1 The Ph.D. Program for Translational Medicine, College of Medical Science and Technology, Taipei Medical University, 11031 Taipei, Taiwan; 2 Master Program for Clinical Pharmacogenomics and Pharmacoproteomics, Taipei Medical University, 11031 Taipei, Taiwan; 3 TMU Research Center of Cancer Translational Medicine, Taipei Medical University, 11031 Taipei, Taiwan; 4 Ph.D. Program of Cancer Biology and Drug Discovery, Taipei Medical University, 11031 Taipei, Taiwan; 5 Integrated Laboratory, Center of Translational Medicine, Taipei Medical University, 11031 Taipei, Taiwan; 6 Core Facility, Taipei Medical University, 11031 Taipei, Taiwan; 7 Chemical Engineering, California Institute of Technology, Pasadena, CA 91125 USA; 8 Graduate Institute of Cancer Biology and Drug Discovery, Taipei Medical University, 11031 Taipei, Taiwan; 9 Cancer Center, Taipei Municipal WanFang Hospital, 11696 Taipei, Taiwan

**Keywords:** Nanoparticle, Camptothecin, Immune responses, Brain tumor

## Abstract

**Introduction:**

Efficacy and safety are critical concerns when designing drug carriers. Nanoparticles are a particular type of carrier that has gained recent attention in cancer therapeutics.

**Methods:**

In this study, we assess the safety profile of IT-101, a nanoparticle formed by self-assembly of camptothecin (CPT) conjugated cyclodextrin-based polymers. IT-101 delivers CPT to target cancer cells in animal models of numerous human cancers and in humans. Previous data from preclinical and clinical trials indicate that IT-101 has no notable immunological side effects. However, there have been no published studies focused on evaluating the effects of IT-101 on host immune systems.

**Results:**

In this work, we demonstrate that IT-101 diminished initial host immune response following first injection of the nanopharmaceutical and induced NK cell activation and T cell proliferation upon further IT-101 exposure. Additionally, IT-101 could attenuate tumor growth more efficiently than CPT treatment only.

**Conclusions:**

Drugs administration in whole-body circulation may lead to poorly bioavailable in central nervous system and often has toxic effects on peripheral tissues. Conjugated with cyclodextrin-based polymers not only reduce adverse effects but also modulate the immune responses to elevate drug efficacy. These immune responses may potentially facilitate actions of immune blockage, such as PD1/PDL1 in cancer treatment.

## Background

The immune system is responsible for much of the body’s systemic response to and modulation in internal and external environmental stimuli. All foreign entities, biological or chemical, may interact with the immune system to cause localized and/or systemic immune responses. Accordingly, entities that can stimulate or suppress the immune system warrant investigation. Recently, nanoparticles have been investigated as an emerging medical application, particularly for drug delivery, and claims of their ability to bypass attack by host immune systems, or activating immune responses have appeared [1–5]. Therefore, understanding the immune response induced by engineered nanoparticles is a critical priority when evaluating preclinical or clinical trials in the future [6–8].

IT-101 is an engineered nanoparticle consisting of cyclodextrin-based polymer conjugate of camptothecin (CPT) that is formed by self-assembly [3]. This nanoparticle is highly soluble, has long circulating half-life in mammals and selectively targets solid tumors [9, 10]. IT-101 has been used to target and inhibit tumor growth, and has successfully been translated from animals (preclinical) to human (clinical) studies. IT-101 has also been shown to prolong the survival rate xenograft-bearing mice with a wide variety of human tumor types [11] including human B cell lymphoma [12] and intracranial U87MG glioma-bearing nude mice [13]. Additionally, there are noticeable similarities in pharmacokinetic (PK) data obtained from rats, dogs, and humans [14], demonstrating that IT-101 is translatable from animals to human. The first phase 1/2a clinical trial indicated the safety, pharmacokinetics and efficacy of IT-101 treatment [14, 15]. Clinical evaluation of IT-101-like nanoparticles continue in Phase II trials (name changed to CRLX101 and more recently NLG207).

Nanoparticles may be able to provide high-efficiency solutions to delivering different kinds of drugs to the target sites in mammals, and thus, whether nanoparticles can synergistically or antagonistically affect immune responses is an essential feature that requires further attention [16, 17]. Although previous reports have indicated that cyclodextrins alone do not elicit immune responses [18], to date, there has not been detailed studies on this topic for cyclodextrin containing polymers, as well as no reports about the effects of IT-101 on host immune systems. To understand the immune effects of IT-101, we systematically analyzed the changes in lymphoid and myeloid cell populations related to innate and adaptive immune responses and the subsequent immune effects, such as cytokine secretion into circulation upon dosing IT-101 in mice (we maintain the IT-101 nomenclature as the materials used is more consistent with the properties reported for IT-101 [3, 9–11].

## Methods

### Animals

The inbred FVB strain mouse is widely applied in cancer research. FVB wild-type mice were bred in a specific-pathogen-free facility. The mouse model with spontaneous brain tumor, F1B-Taq transgenic mouse, was obtained from Dr. Ing-Ming Chiu in National Health Research Institutes (NHRI) [19]. Euthanasia was performed using CO_2_ inhalation. The animal protocol was approved by the Institutional Animal Care and User Committee of National Defense Medical Center.

### IT-101 nanoparticle

IT-101 is a polymeric nanoparticle composed of CPT covalently conjugated to the linear, cyclodextrin-polyethylene glycol (CD-PEG) copolymer, that self-assembles into nanoparticles. The synthesis and properties of IT-101 have been reported previously [3, 9–11]. As a positive control, CPT was dissolved in PBS with 2% dimethyl sulfoxide (DMSO, Sigma). Both IT-101 and CPT were inoculated at 10 mg/kg (for IT-101 the amount is the CPT equivalents dosed) by intravenous (i.v.) and intraperitoneal (i.p.) injection, respectively, to 2- and 4-month-old mice. PBS and 2% DMSO in PBS were used as negative controls.

### Flow cytometry

Immune responses were analyzed by flow cytometry. Four-month-old wild-type FVB mice were inoculated by intravenous injection with PBS and IT-101 and intraperitoneal injection with DMSO and CPT, respectively, and sacrificed for experimental manipulation. The cells in peripheral blood, spleen and groin lymph nodes were collected and resuspended in RPMI-1640 medium. After washing twice with stain buffer (BD), cells from spleen and lymph nodes (1 × 10^6^) and 0.1 mL of peripheral blood were conjugated with different antibodies (CD11b, CD11c, CD3, CD4, CD45, CD8a, CD19, F4/80, Gr-1, MHCII, NK1.1). All cells were passed through 100 μm pore-size nylon mesh and analyzed by BD FACSVerse flow cytometry.

### Intracellular cytokine staining (ICS)

FVB mice were sacrificed after 9 days of i.v injection. Peripheral blood was incubated with PMA (20 ng/ml) plus ionomycin (1 μl /ml) (BioLegend Cat. No. 423301) and monensin (BioLegend Cat. No. 420701) at 37 °C. After 6 h, lysed cells and stained surface marker Ab for T and NK cells (CD3 and NK1.1) for 20 mins at room temperature, and then treated with fixation buffer (BioLegend Cat. No. 420801) for 20 mins. Cells incubated in Intracellular Staining Perm Wash Buffer (BioLegend Cat. No. 421002) for 15 mins, and then performed intracellular staining for TNF-α, IFN-γ for 30 mins. The data were detected by Flow cytometer (BD FACSVerse).

### Cytokine assay

We used RayBio C-Series Mouse Cytokine Antibody Array Kit (RayBiotech) to evaluate expression levels of various cytokines. The antibody array was carefully removed from the plastic packaging, and each membrane was placed into a well of the incubation tray with 2 mL of blocking buffer for 30 mins at room temperature. Blocking buffer was then removed from each well, and the wells were incubated with serum or plasma at 4 °C overnight. Following aspiration of the samples from each well, 1 mL of detection antibody was applied for 2 h at room temperature followed by 2 mL of HRP-Streptavidin Concentrate for 2 h at room temperature. Three washes were performed between each incubation step. Detection buffer was added onto each membrane and incubated for 2 min at room temperature. Each array membranes were observed and analyzed by a chemiluminescence imaging system. Calculated data values higher than 1.5 or lower than 0.6 indicated significantly difference between two groups, respectively.

### ELISA

FVB mice were injected with IT-101 or solvent (PBS buffer) intravenously; serum was harvested after 9 days of treatment. Each group comprised of three mice. TNFα and IFN-γ levels were measured by the LEGEND Max ELISA Kit with Precoated Plates (430,907 and 430,807, Biolegend). IL-10 was measured by the Interleukin-10 Mouse ELISA Kit (ab100697). The results were analyzed using a Synergy H4 Reader (Bio Tek Instruments).

### Histopathological analysis

After mice were anesthetized with 0.4–0.6 mL avertin, we harvested brain by perfusion with 10 mL 4% paraformaldehyde and fixed half-brain by 10% formalin. Paraffin-embedded samples slides were subjected to hematoxylin and eosin (H&E) staining. Tissue sections were also subjected to immunohistochemistry (IHC). The IHC staining was performed by incubating sections with primary antibodies targeting CD3 (ab5690, 1:100, abcam) for 18–24 h at 4 °C, and then performed using biotinylated secondary antibodies and an LSAB Kit (DakoCytomation). Sections were observed by microscopy (Olympus IX73). Tumor size was measured by software (SPOT Imaging).

### Statistical analysis

Student’s *t*-test was performed using GraphPad Prism5 software with significance set at *P* < 0.05. Data are presented as the mean ± SD for all treatments.

## Results

### Innate immune response modulated by IT-101

**Fig. 1:**
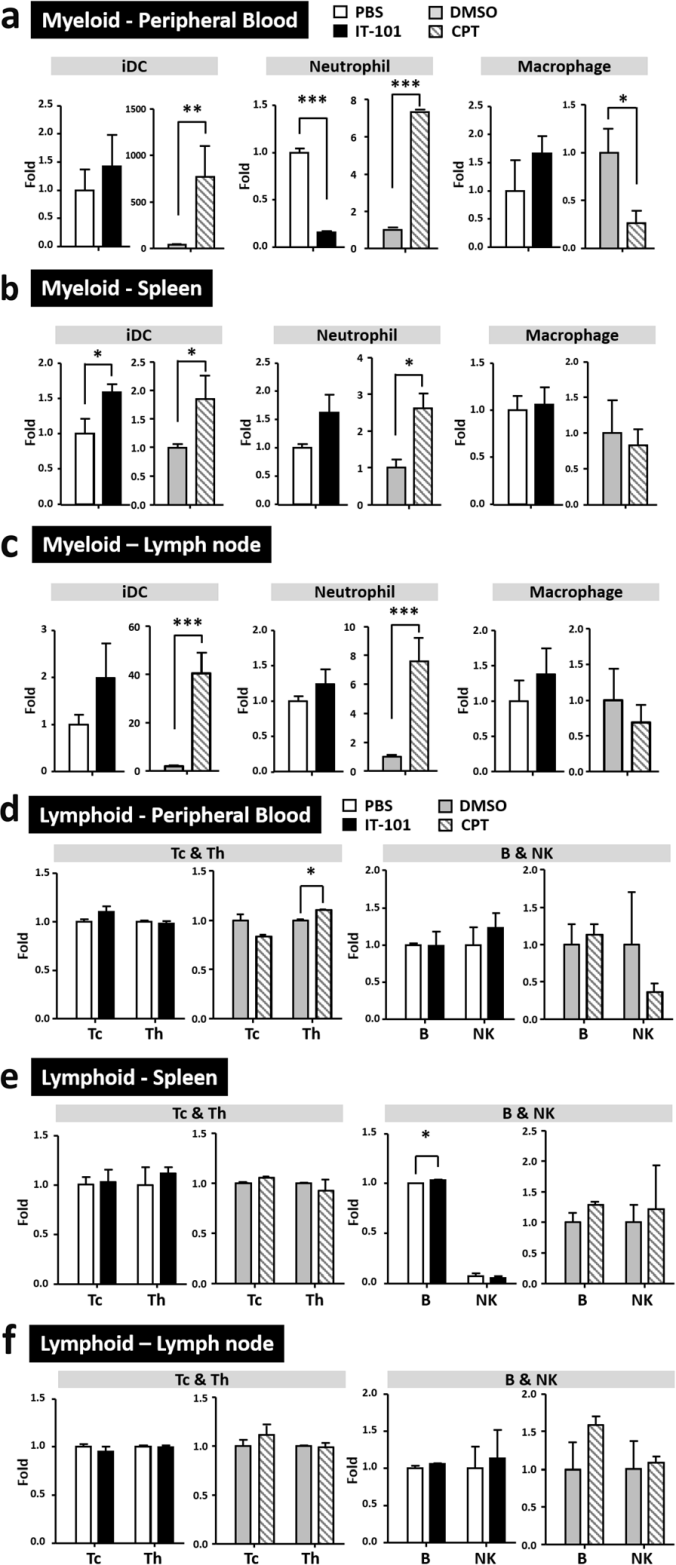
Innate immune responses at 16 h post induction by the nanoparticle IT-101. (**a**)-(**c**) Changes in the proportions of cells in the myeloid cell lineage, including iDCs, neutrophils and macrophages, in peripheral blood, spleen and groin lymph nodes. (**d**)-(**f**) Changes in the proportions of cells in the lymphoid cell lineage, including Tc, Th, B and NK cells, in peripheral blood, spleen and groin lymph nodes. Number of mice per group: 3–6 mice. The results are presented as the mean ± SD. *p < 0.05; **p < 0.005; ***p < 0.0001

To detect the natural immune response in mice treated with IT-101 and CPT, the mice were inoculated and analyzed 16 h later (Additional file 1: Fig. S1a). Vehicles for both agents were used as controls, namely PBS for IT-101 and DMSO for CPT. We analyzed the proportion of leukocyte subtypes, as described in Additional file 1: Fig. S1b, based on their major surface markers. We observed lymphoid and myeloid cell lineages in peripheral blood, spleen and groin lymph nodes. We analyzed myeloid cell populations, such as immature dendritic cells (iDCs), neutrophils and macrophages. After CPT treatment, iDC and neutrophil proportion in all three locations were dramatically increased; meanwhile, the population of macrophages was decreased in the circulation (Fig. 1a-c). Additionally, the proportion of neutrophils was significantly decreased in the peripheral blood of mice after IT-101 treatment. No noticeable difference in other myeloid cell subtypes in peripheral lymph organs was observed (Fig. 1a-c; Additional file 1: Fig. S2). The subtypes of lymphoid cells included cytotoxic T lymphocytes (Tc cells), helper T lymphocytes (Th cells), B lymphocytes (B cells) and natural killer cells (NK cells). There was no notable difference in the lymphoid cell populations among mice treated with IT-101, CPT and their controls (Fig. 1d-f). Overall our data suggest that IT-101 reduces a select myeloid cell population and this may modulate host immune response when injected in FVB mice.

### Cytokine analysis after one-dose of IT-101: inhibition of inflammatory responses

**Fig. 2:**
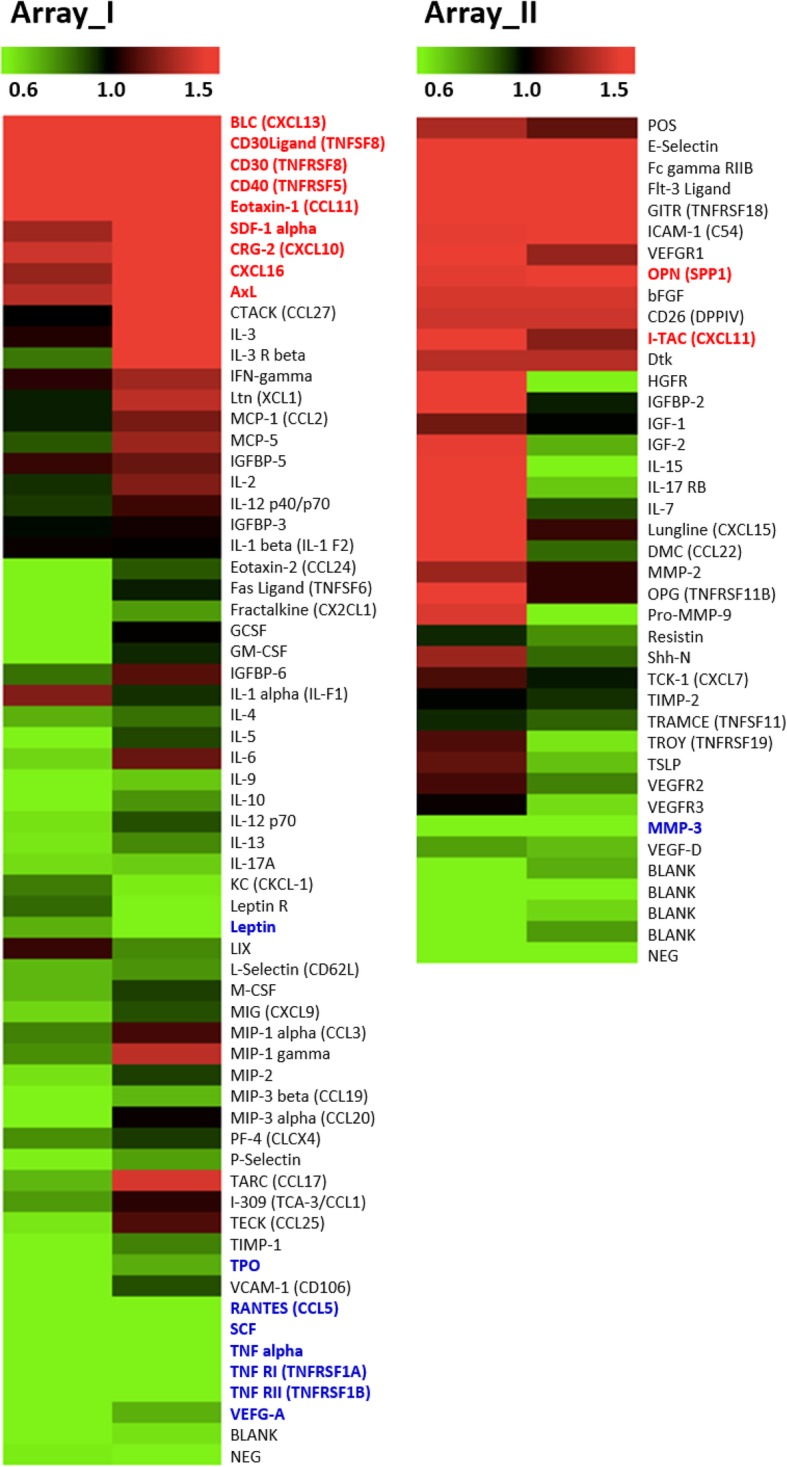
Changes in cytokine expression after IT-101 nanoparticle treatment. Heat map showing the increase or decrease in cytokine levels after IT-101 nanoparticle treatment. RayBio C-Series Mouse Cytokine Antibody Array Kit (RayBiotech) was used to evaluate expression levels of various cytokines. Array I is the probed membrane for RayBio C-Series Mouse Cytokine Antibody Array C3 (monitoring 62 mouse proteins). Array II is the probed membrane for RayBio C-Series Mouse Cytokine Antibody Array C4(monitoring 34 mouse proteins)

We examined the expression levels of various cytokines in peripheral blood following IT-101 exposure to determine potential changes in host inflammatory response. Serum collected from FVB mice treated with IT-101 and control (PBS) was analyzed by independent cytokine assays (Additional file 1: Fig. S3). After IT-101 treatment, the expression levels of several chemokines were increased; these chemokines include CXCL11 (I-TAC), CXCL12 (SDF-1), CXCL13 (BLC), CXCL10 (CRG-2) and CXCL16, that attract NK cells and probably other cells, as well as soluble CD30 ligand (sCD30L; TNFSF8), soluble CD30 (sCD30; TNFRSF8), and soluble CD40 (sCD40; TNFRSF5), that interfere with T or B cell functions (Fig. 2; Table 1). In addition to avoiding severe inflammation, one injection of IT-101 decreased the expression levels of TNFα and its receptors, TNFRI (TNFRSF1A) and TNFRII (TNFRSF1B) (Fig. 2; Table 1). We noted that IT-101 treatment seemed to inhibit inflammation and that further downstream immune responses were inhibited following the first immunization with IT-101. We also examined the expression levels of various cytokines in peripheral blood following CPT exposure for analyzing the potential differences between IT-101 and CPT in host inflammatory response. Serum collected from FVB mice treated with CPT and control (DMSO) was analyzed by independent cytokine assays (Additional file 1: Fig. S4). After CPT treatment alone, trend change of several cytokines were similar to samples with IT-101 treatment, such as Leptin, RANTES (CCL5), SCF, CXCL11 (I-TAC), CXCL12 (SDF-1) and OPN; meanwhile, trend change of some cytokines were opposite to samples with IT-101 treatment, such as CD30, CD40, CXCL10 (CRG-2), CXCL16, Eotaxin-1 and MMP-3. Except above mentioned, CPT treatment also modulated significant changes of various cytokines or soluble factors, especially elevating CXCL, CCL, MMP, growth factor and several immune-regulated factors. Altogether, our data suggest that IT-101 seems to provide a barrier avoiding the severe inflammatory responses after first immunization with CPT.

**Table 1:**
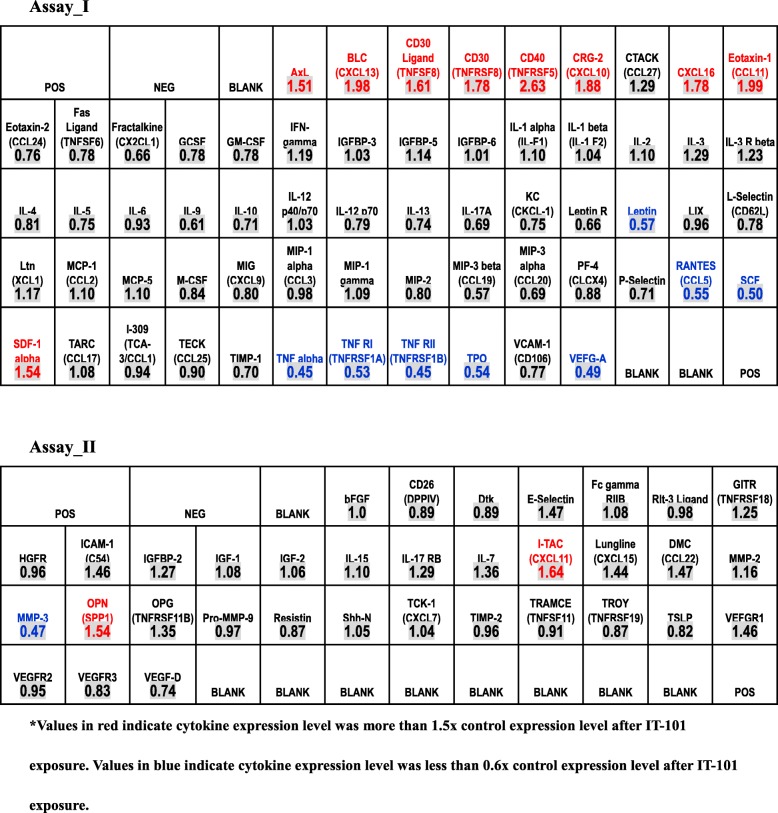
Quantified data for expression levels of cytokines and other secreted factors. Assay_I. *Values in red indicate cytokine expression level was more than 1.5x control expression level after IT-101 exposure. Values in blue indicate cytokine expression level was less than 0.6x control expression level after IT-101 exposure

### Adaptive immune response induced by IT-101: increases of NK cell and T cell populations

**Fig. 3:**
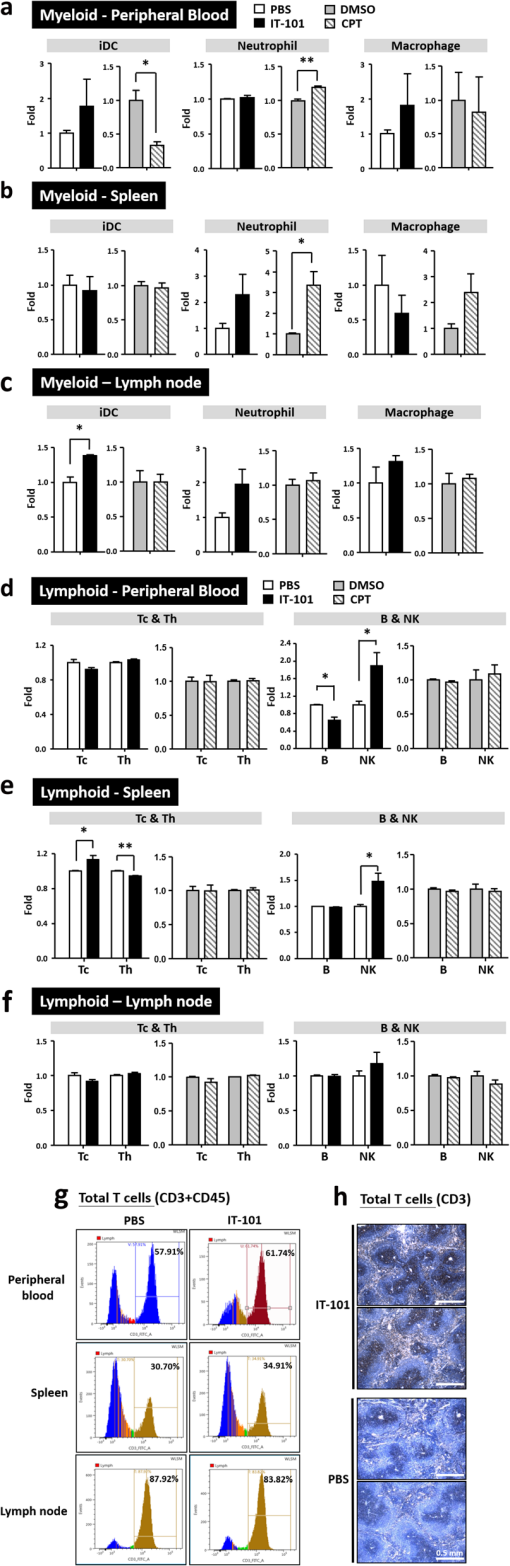
Adaptive immune responses after the second stimulation by the nanoparticle IT-101. (**a**)-(**c**) Changes in the proportions of cells in the myeloid cell lineage, including iDCs, neutrophils and macrophages, in peripheral blood, spleen and groin lymph nodes. (**d**)-(**f**) Changes in the proportions of cells in the lymphoid cell lineage, including Tc, Th, B and NK cells, in peripheral blood, spleen and groin lymph nodes. (**g**) Quantification of total T cells, labeled with CD3 and CD45, in peripheral blood, spleen and lymph node. (**h**) Increased T lymphocytes were observed in spleen after IT-101 treatment. Number of mice per group: 3–6 mice. The results are presented as the mean ± SD. *p < 0.05; **p < 0.005; ***p < 0.0001

In addition to innate immunity, we also measured indications of the adaptive immune responses following IT-101- and CPT-boosted treatment. Reinjection of CPT elevated the proportion of neutrophils in blood circulation and spleen, that caused a decrease in immature DC cells in peripheral blood (Fig. 3a-c; Additional file 1: Fig. S5a-b). Intriguingly, in peripheral blood and spleen, IT-101-boosted treatment enhanced the NK cells and slightly increased DCs in peripheral blood and lymph node, as well as increased the total T cell proportion and the Tc cells. (Fig. 3d-g; Additional file 1: Fig. S5c-e).

**Fig. 4:**
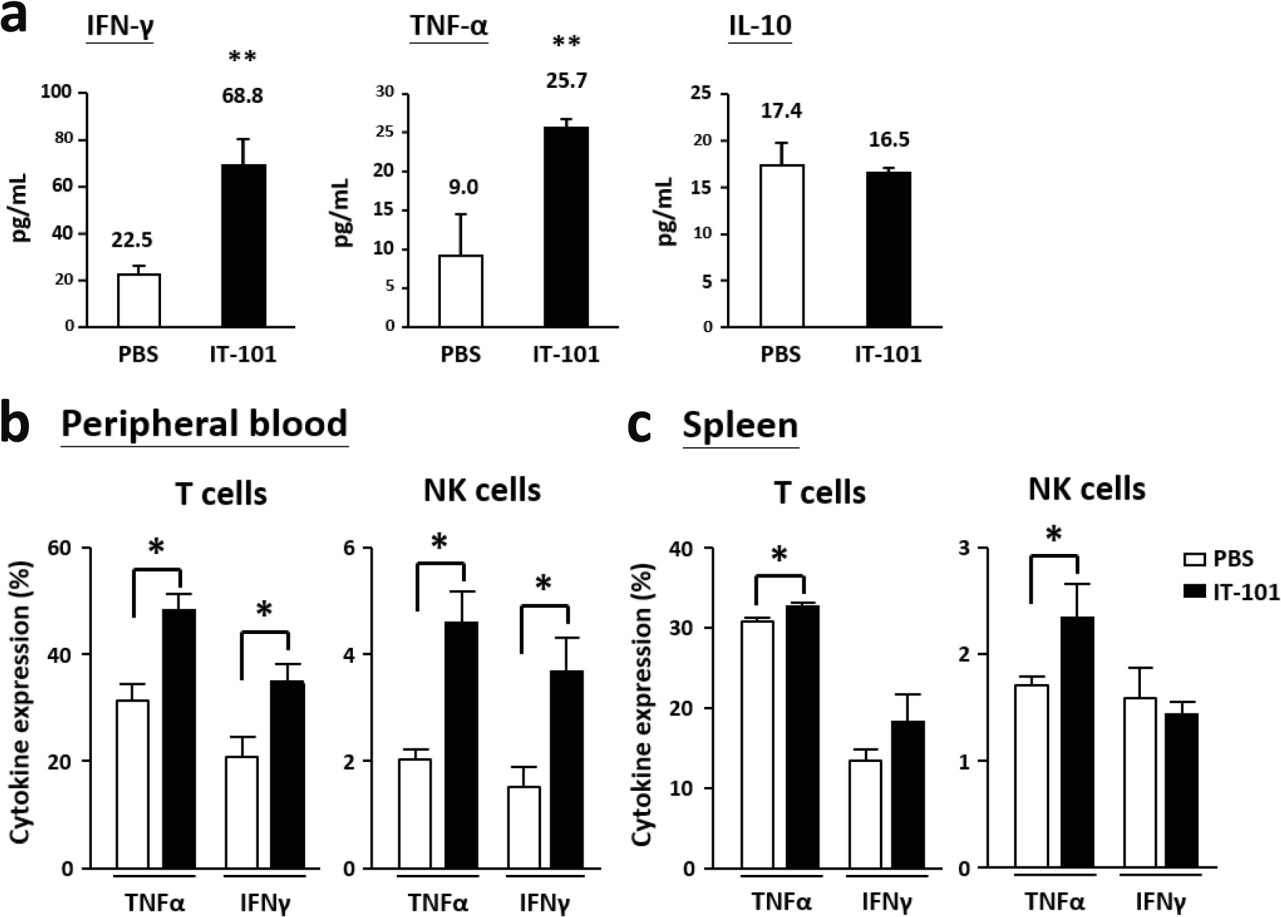
The expression levels of activated cytokines and inhibitory cytokines after IT-101 treatment. (**a**) The expression levels of IFN-γ, TNF-α and IL-10 were detected after IT-101 treatment. (**b**) Higher proportion of TNF-α- and IFN-γ-secreted T cells and NK cells were observed in peripheral blood after IT-101 treatments. (**c**) Higher proportion of TNF-α-secreted T cells and NK cells were observed in spleen. Mouse number per group, 3 mice. The results are presented as the mean ± SD. *p < 0.05; **p < 0.0005

We summarized the effects of IT-101 and CPT on different immune cell populations after 16 (one dose) and 216 (one dose with booster doses) hours of treatment (Table 2). The single-dose CPT treatment significantly increased the proportions of neutrophils and immature DCs, both of which mainly participate in the innate immune response. IT-101 treatment with a single dose plus booster doses markedly increased the proportion of NK cells and total T cells. These results lead us to determine the expression levels of NK cell-secreted cytokines in blood circulation. Indeed, levels of the proinflammatory cytokine TNFα and the T cell-activating cytokine INF-γ were noticeably increased; meanwhile, the immune-inhibitory cytokine IL-10 showed no significant difference between the IT-101- and control (PBS)-treated groups (Fig. 4a). TNFα-positive T and NK cells were detected in peripheral blood and spleen, and increased INF-γ-positive T cells were observed in peripheral blood after IT-101 treatment. (Fig. 4b-c). Therefore, we observed that IT-101 enhances the proportion and activation of NK cells to facilitate T cell activation and dendritic cell migration, rather than initiating innate immune responses.

**Table 2:**
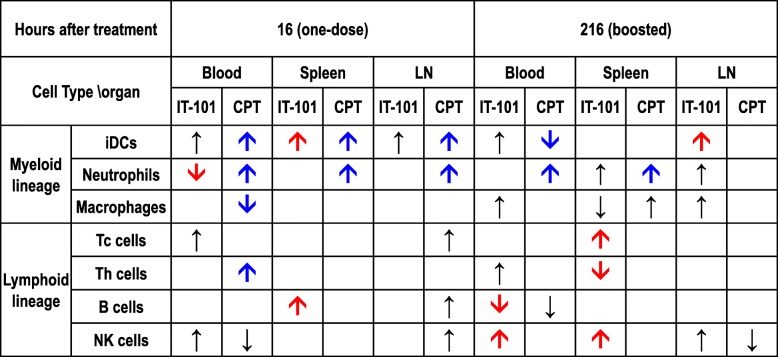
Summary for innate and adaptive immune responses after IT-101 and CPT treatment

### IT-101 efficiently attenuated tumor growth in mice

**Fig. 5:**
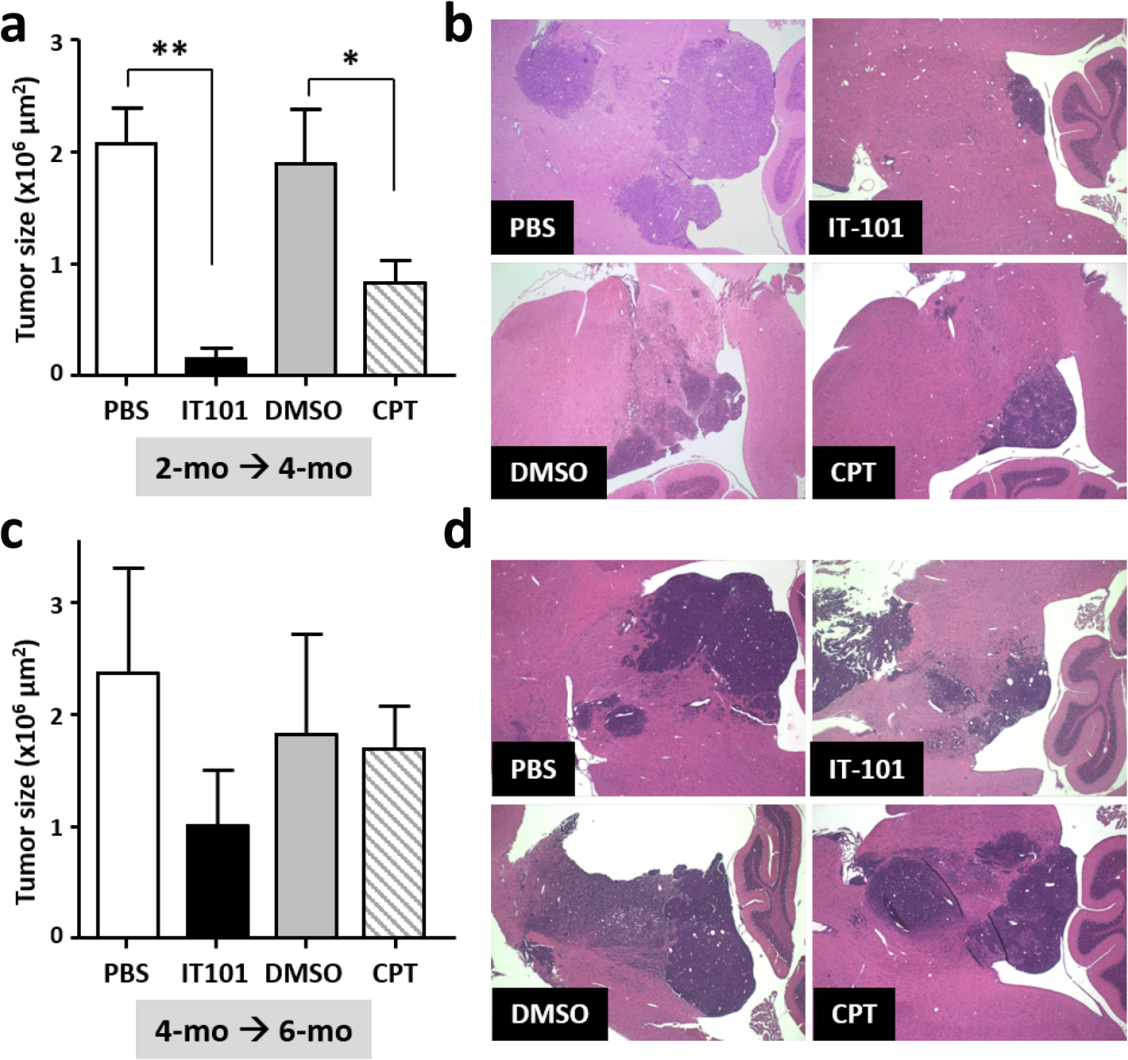
IT-101 attenuated the brain tumor growth at early stage of tumorigenesis. **(a) (b)** 2-month-old F1B transgenic mice were treated with IT-101 and PBS respectively by intravenous injection, one dose with booster doses. After two months ago, the tumor size was measured using histopathological data. (**c**) (**d**) 4-month-old F1B transgenic mice were treated with IT-101 and PBS respectively by Intraperitoneal injection, one dose with booster doses. After two months ago, the tumor size was measured using histopathological data. Mouse number per group, 3–5 mice. The results are presented as the mean ± SD. *p < 0.05; **p < 0.005

To verify whether cyclodextrin-based polymer can facilitate CPT to inhibit tumor growth, we treated a mouse model bearing spontaneous brain tumor with IT-101, CPT and their control solvents respectively. The average lifespan of F1B transgenic mice was around 6 months. More than 50% of 2-month-old and almost all of 4-month-old F1B transgenic mice had obvious tumors in tegmental area of brain (Additional file 1: Fig. S6). CPT treatment at early stage of tumorigenesis (2 months of age) could significantly attenuate the tumor growth, and notably IT-101 treatment had higher inhibitory effect on tumorigenesis (Fig. 5a-b). However, at late stage of tumorigenesis (4 months of age), IT-101 also had a chance to attenuate the tumor growth, and only CPT treatment had no significant effect on tumorigenesis (Fig. 5c-d).

## Discussion

Cancer immunotherapy is becoming commonplace for standard cancer treatment. However, solid tumors can shape an immunosuppressive tumor environment to bypass anticancer immune responses. Recent studies in translational research have highlighted nanoparticles as an emerging treatment modality for cancer. Various biomaterial-based nanoparticles have been applied to improve therapeutic efficacy, specificity, and stability. Nanoparticles can have prolonged half-life to enable the circulation of nanoparticle-delivered drugs to the whole body. Moreover, nanoparticles have the potential advantage of targeting specific cell types within target organs. However, there have been concerns regarding the safety of nanoparticles; thus, the toxicity, hazards, and immune responses related to nanoparticles have become safety issues of importance [6, 20].

**Fig. 6:**
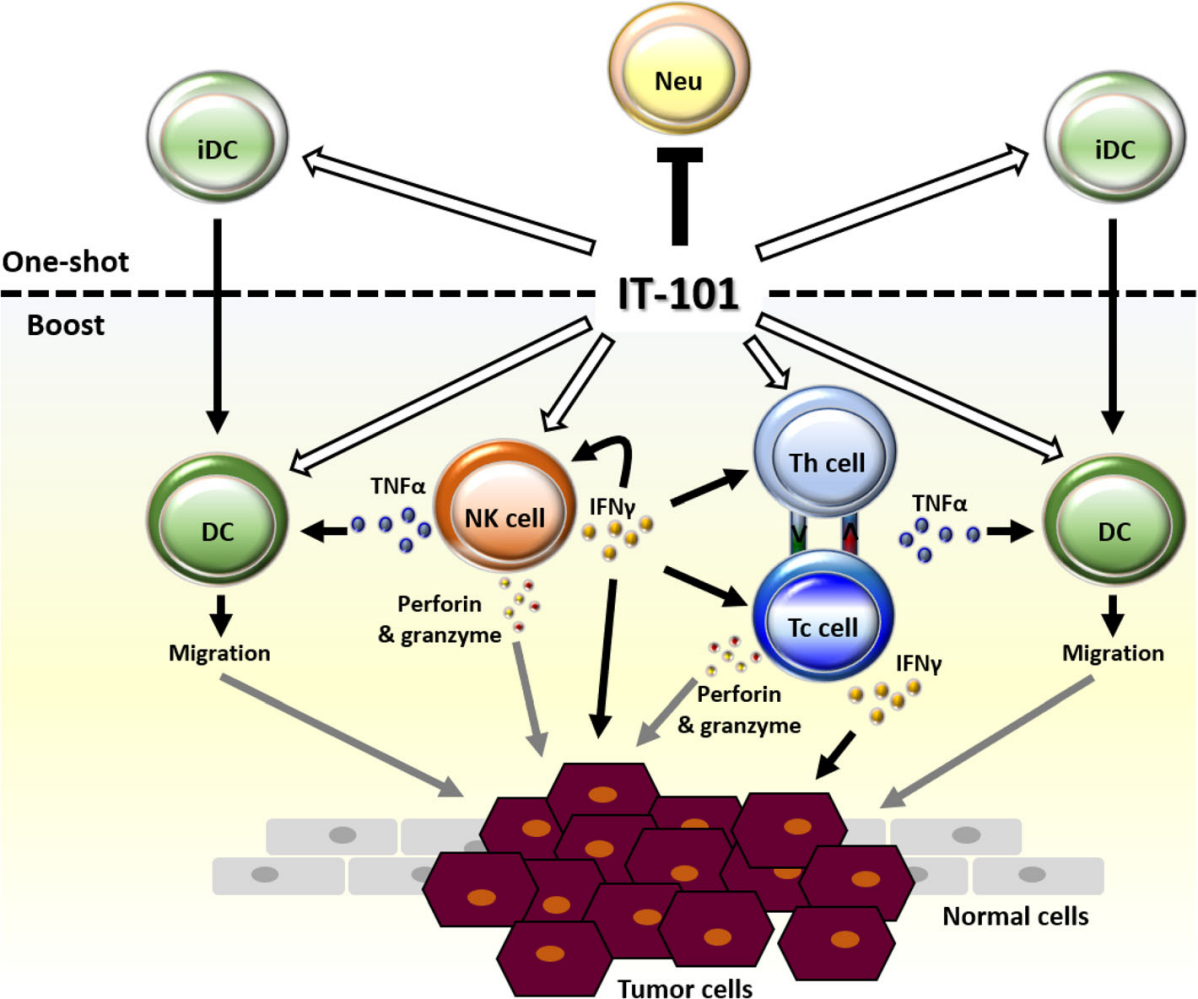
IT-101 induced activation of NK cells, which facilitates other immune cells to attack tumors. First immunization with IT-101 (CPT conjugated with CD-PEG) modulated the populations of neutrophil (Neu) and immature dendritic cells (iDC). Boosting with IT-101 induced affected NK cells to secrete IFNγ and TNFα. IFNγ promotes Th and Tc cell activation, and TNFα induces dendritic cell (DC) migration. Both NK and Tc cells generate and secrete perforin and granzyme to weaken and destroy tumor cells. Black lines indicate the results we suggested in this study. Gray lines indicate the results we predicted based on our results

Here, we demonstrated that IT-101, a nanoparticle drug, did not induce non-specific inflammation, but accurately altered the status of immune surveillance. First immunization of IT-101 modulated an increase of immature dendritic cells in lymphoid organs and a decrease of neutrophils in peripheral circulation, companying with several cytokines or specific factors induction. As previous reports, IT-101 has long PK and better area under the curve (AUC) to continuously those immune responses [14]. Subsequently, boost injection of IT-101 induced effected NK cells and T cells to secrete IFNγ and TNFα. TNFα might induce dendritic cell migration, and IFNγ can promote Th and Tc cell activation. These effects may enhance the function of effected NK cells and exert anti-tumor function (Fig. 6). Both NK and Tc cells can generate and secrete perforins and granzymes to weaken and destroy tumor cells [21, 22]. Thus, the action of CPT may work together with these effects to inhibit tumor growth.

After one-dose injection, CPT induced complicated immune responses comparing to IT-101 treatment. Our data showed that iDC and neutrophil proportion in all three locations were obviously increased in CPT-treated group, and indeed, the factors, which are responsible for recruiting, activating and mediating activity for iDCs and neutrophils, were elevated expressed in peripheral lymphoid organs. These soluble factors include GM-CSF, MMP, TNF, IGF, IL-7, IL-15, IL-17, resistin, CCL2, CCL22, CXCL7, VEGF, TSLP, TIMP and other undetermined factors. However, CPT conjugated cyclodextrin-based polymers could avoid from side effects of complicated immune responses, and modulate the adaptive immune responses after boosting.

Although some proinflammatory factors or neutrophil-attracting factors, such as MMP3, CCL5, leptin and VEGF-A, were decreased in the IT-101-treated group, other factors like sCD30, sCD30L, sCD40 and sCD40L (that are reported inflammatory markers in cancer patients), were elevated in the circulation of IT-101-treated mice. In ovarian cancer patients, an increase in sCD30, sCD30L and sCD40 could be early diagnostic indicators, and all of these factors may impair apoptosis and interrupt the immune response [23]. The expression levels of sCD30, as well as sCD30L, in circulation could also be used as a prognostic marker for Hodgkin lymphoma [24–26]. High levels of sCD40 have been found associated with advanced pathogenesis and poor prognosis in non-small cell lung cancer patients [27]. Similarly, reports have showed high levels of sCD40L in patients with neoplasia and nasopharyngeal carcinoma (NPC); hence, sCD40L might be a prognostic biomarker for NPC [28, 29]. Additionally, sCD40L could be a diagnostic marker for hepatitis C virus-related hepatocellular carcinoma [30]. The CD40/CD154 interaction plays critical functions in humoral and cellular immune responses. Elevated levels of the soluble form of CD40 (sCD40) have been observed in patients in uremic or hemodialyzed situations; of note, sCD40 is able to inhibit immunoglobulin generation from CD154-induced activated B cells [31].

Previous reports have indicated that specific antibodies against PEG-coated liposomes could be generated to eliminate PEG-coated liposomes in circulation throughout the whole body; however, accelerated blood clearance caused by PEGylated liposomes was demonstrated to be due to doxorubicin encapsulation and a high-dose first injection [32, 33]. These antibodies have high potential to affect the safety and efficiency of therapeutics. However, after repeated injections of IT-101, we detected no notable increase in the proportion of B lymphocytes and T helper cells. We therefore suggest that IT-101 injection has no or low humoral immunogenicity.

A recent study revealed local injury promotes activated leukocyte infiltration and that these leukocytes further secrete chemokines to induce activated NK cell recruitment [34]. CXCR3 and CXCR4 are primarily expressed on activated NK cells. CXCR3 expressed on NK cells (CD56^high^CD27 ^high^) facilitates enhanced proliferation and cytokine production [35]. Various organ/tissue-specific diseases caused by increased inflammation, such as chronic hepatitis C virus infection, biliary cirrhosis, psoriatic and osteoarthritis, attract NK cells via the CXCR3/CXCL10 axis [36–39]; meanwhile, CXCL11-targeted CXCR3 promotes antitumor responses [40]. The CXCR4/CXCL12 axis play an important role in NK cell development in adults [41]. Rather than assisting NK-mediated immune surveillance against tumor formation and metastasis, some reports have indicated that CXCR3- and CXCR4-mediated attraction of NK cells may impede immune responses [42, 43]. In our study, CXCL11 and CXCL12, whose secretion by peripheral blood leukocytes was increased after IT-101 treatment, interacted with CXCR3 and CXCR4, respectively, further increasing the proportion of activated NK cells after repeat injections and indicating that IT-101 nanoparticles could facilitate the initiation of immune responses through chemotactic axes.

Here, we found that IT-101 could effectively bypass the induction of innate immune responses and increase NK cell activation after boost injection. NK cells have been reported in humans and mice to antagonize virus infection and tumor immunosurveillance [44]. NK cells are innate cytotoxic lymphocytes that kill infected cells or tumor cells via secreting lytic granules containing perforin and apoptosis-inducing granzymes, and release cytokines or secreted factors for inflammation and immunoregulation [45, 46]. In the presence of stressed cells, DC maturation is promoted by NK cell-derived IFN-γ and TNF-α. Subsequently, NK cells secrete IFN-γ to activate T cells and macrophages and, in contrast, secrete IL-10 to dampen the functions of T cells and macrophages. Therefore, NK cells facilitate and enhance the antigen-presenting capacity of immune cells and further regulate immune cells by secreting different cytokines, as NK cells participate in both innate and adaptive immunity [47]. In humans and mice, NK cells are recruited into draining lymph nodes and promote an adaptive immune response through the CXCR3-CXCL10 axis; the attracted NK cells then provide IFN-γ, which is necessary for dendritic cell modulation and T helper cell polarization [48]. IFN-γ and TNF-α stimulate various cells to induce CXCL10 elevation, resulting in recruitment of Type 1 T helper cells [49]. Data from our study suggests IT-101 has the potential to activate NK cells to inhibit tumor spread and induce subsequent immunity by secreting activated cytokines such as IFN-γ and TNF-α.

## Conclusions

Based on our findings, boost injection of IT-101 could regulate NK cell activation and T cell proliferation; and meanwhile, cyclodextrin-based polymer could facilitate CPT to inhibit tumor growth at early stage of tumorigenesis. CPT could be transported into brain by crossing the blood-brain barrier (BBB), but as we known, cyclodextrin-based polymer may not pass through the BBB. Therefore, IT-101, which deliver CPT closing to the brain, may have no adverse effect directly on central nervous system, and focus on modulating the immune responses in peripherals. Conjugated with cyclodextrin-based polymer may reduce the adverse effects of CPT on peripheral tissues and efficiently elevate bioavailability of CPT in brain. We expect IT-101 may be used with other anti-cancer drugs to achieve synergistic suppression of tumor growth. Recent clinical studies highlight the efficacy of PD-1 and PD-L1 duo inhibition as a promising anti-tumor therapeutic strategy [50, 51]. PD-1/PD-L1 inhibits T cell survival, proliferation, cytotoxicity and cytokine secretion, protection of tumors from attacking of cytotoxic T cells, and induction of cell death in tumor-specific T lymphocytes [52–55]. Immune checkpoint inhibitors (antibodies against PD-1/PD-L1 or CLTA-4) modulate the host’s own immune responses to attack tumor tissues, even the brain metastases [56, 57]. Hence, combined treatment of IT-101 with PD-1/PD-L1 blocker may be an interesting therapeutic strategy, since IT-101 treatment can induce effected NK cells to facilitate T cell activation, and blocking of PD-1/PD-L1 can subsequently promote T cell proliferation and activation. Upon breakdown of immune surveillance of tumors by IT-101, CPT can then target and inhibit tumor cell proliferation.

## Supplementary information

**Additional file 1: Fig. S1.** Working schemes for immunological analysis of nanoparticle IT-101 in mice. **Fig. S2.** Significant changes after 16 h IT-101 treatment were presented by dot plots. **Fig. S3.** Cytokine assay. **Fig. S4.** Changes of cytokine expression after CPT treatment. **Fig. S5.** Significant changes after 216 h treatment were presented by dot plots. **Fig. S6.** Early stage of tumorigenesis in F1B transgenic mice.

## Data Availability

All data and materials supporting the conclusion of this study have been included with the article and the additional information.

## Abbreviations

BBB: blood-brain barrier
CCL: Chemokine (C-C motif) ligand
CD-PEG: cyclodextrin-polyethylene glycol
CPT: camptothecin
CTLA-4: cytotoxic T-lymphocyte–associated antigen 4
CXCL: C-X-C motif chemokine
CXCR: CXC chemokine receptors
iDCs: Immature dendritic cells
IFNγ: Interferon γ
MMP: Matrix metalloproteinase
NK cells: Natural killer cells
PD-1: Programmed cell death 1
PD-L1: Programmed cell death 1 ligand 1
PK: Pharmacokinetic
SCF: Stem cell factor
Tc cells: Cytotoxic T lymphocytes
Th cells: Helper T lymphocytes
TNFRSF: Tumor necrosis factor receptor superfamily member
TNFSF: Tumor necrosis factor superfamily member
TNFα: Tumor necrosis factor α
