# Supplementary material for: Host immune response to anti-cancer camptothecin conjugated cyclodextrin-based polymers
Source: J Biomed Sci. 2019 Oct 23;26:85. doi: 10.1186/s12929-019-0583-0 (PMC6806548; doi:10.1186/s12929-019-0583-0)
Supplement: Supplementary file 1 — Additional file 1: Fig. S1. Working schemes for immunological analysis of nanoparticle IT-101 in mice. Fig. S2. Significant changes after 16 h IT-101 treatment were presented by dot plots. Fig. S3. Cytokine assay. Fig. S4. Changes of cytokine expression after CPT treatment. Fig. S5. Significant changes after 216 h treatment were presented by dot plots. Fig. S6. Early stage of tumorigenesis in F1B transgenic mice. [file 12929_2019_583_MOESM1_ESM.pdf]

Additional file 1

Host immune response to anti-cancer camptothecin conjugated cyclodextrin-based polymers

Yi-Fan Chen[1,2], Yen-Hsin Wang[3], Cing-Syuan Lei[1], Chun A. Changou[4,5,6], Mark E. Davis[7], Yun Yen[3,4]

[1]The Ph.D. Program for Translational Medicine; [2]Master Program for Clinical Pharmacogenomics and Pharmacoproteomics, [3]TMU Research Center of Cancer Translational Medicine, [4]Ph.D. Program of Cancer Biology and Drug Discovery, [5]Integrated Laboratory, Center of Translational Medicine, [6]Core Facility, Taipei Medical University, 11031 Taipei, Taiwan

[7]Chemical Engineering, California Institute of Technology, Pasadena, CA 91125.

Supplemental Figure S1-S6

Supplemental Fig. S1

a

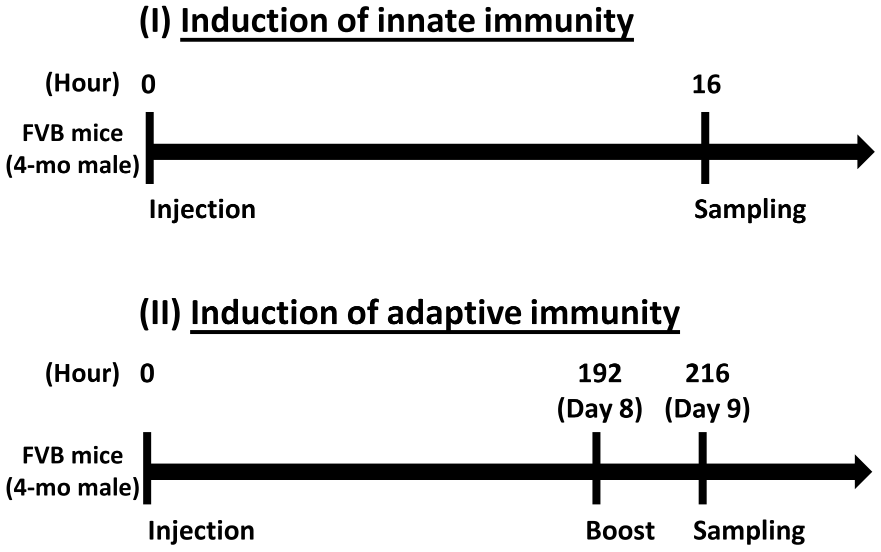

b

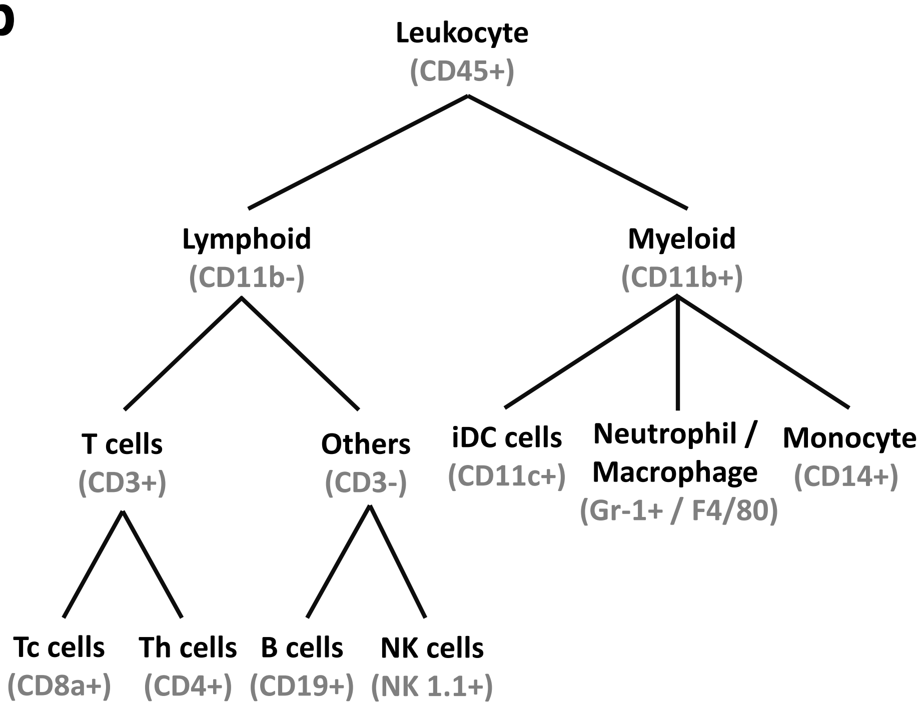

Supplemental Fig. S1. Working schemes for immunological analysis of nanoparticle IT-101 in mice. (a) Schedule for materials delivery and samples harvest. **(b)** Plans for cell collection of lymphoid cell lineage and myeloid cell lineage by flow cytometry.

Supplemental Fig. S2

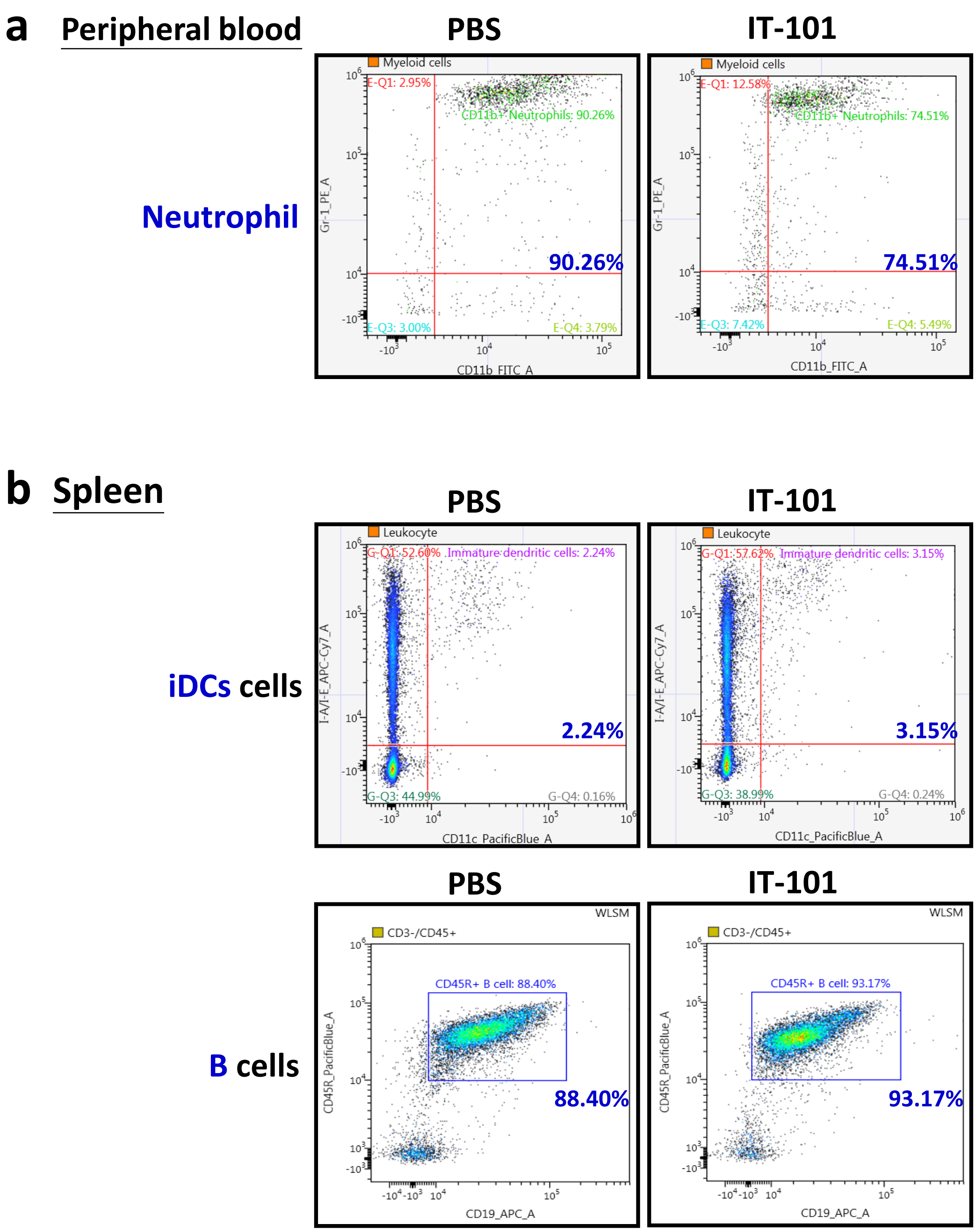

Supplemental Fig. S2. Significant changes after 16h IT-101 treatment were presented by dot plots. Changes of cells populations in peripheral immune system after IT-101 treatment . **(a)** In peripheral blood, the percentage of neutrophils was decreased. **(b)** In spleen, the percentage of B cells and iDCs were increased.

Supplemental Fig. S3

a **Assay_I**

PBS_sample 1 **PBS_sample 2** **IT-101_sample 1** **IT-101_ sample 2**

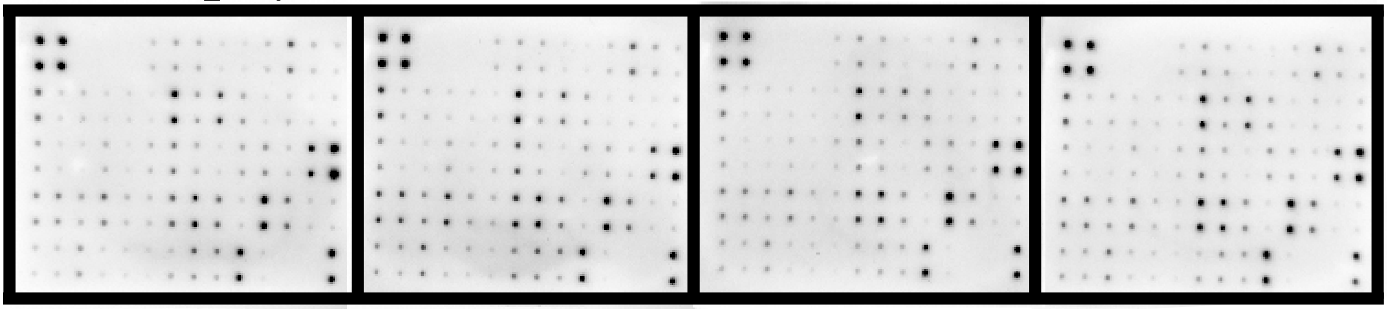

b **Assay_II**

PBS_sample 1 **PBS_sample 2** **IT-101_sample 1** **IT-101_ sample 2**

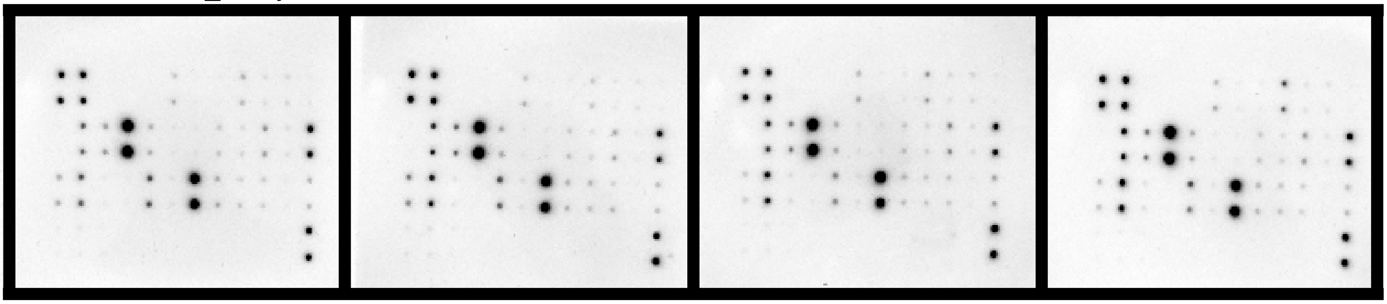

Supplemental Fig. S3. Cytokine assay. (a) Probed membrane for RayBio C-Series Mouse Cytokine Antibody Array C3. **(b)** Probed membrane for RayBio C-Series Mouse Cytokine Antibody Array C4. **(c)** Semi-quantitative detection of 62 mouse proteins in serum using for RayBio C-Series Mouse Cytokine Antibody Array C3. **(d)** Semi-quantitative detection of 34 mouse proteins in serum using for RayBio C-Series Mouse Cytokine Antibody Array C4.

Supplemental Fig. S3_Continued

c Assay_I

IT-101_sample 1

1.00		0.63		0.6	1.32	1.95	1.55	1.66	2.35	1.37	1.00	0.25	1.62
0.60	0.56	0.50	0.52	0.58	1.06	0.98	1.08	0.85	1.21	1.0	0.94	1.05	0.84
0.74	0.56	0.67	0.50	0.59	0.93	0.65	0.64	0.66	0.83	0.86	0.74	1.08	0.73
0.96	0.96	0.89	0.73	0.67	0.82	0.80	0.65	0.39	0.34	0.80	0.62	0.50	0.51
1.27	0.73	0.78	0.64	0.54	29	0.41	0.32	0.32	0.61	0.22	0.11	0.40	0.69

IT-101_ sample 2

1.00		0.54		0.65	1.72	2.00	1.67	1.89	2.92	2.41	1.60	2.33	2.38
0.89	0.96	0.78	1.01	0.95	1.27	1.03	1.16	1.13	0.94	1.01	1.21	1.46	1.57
0.85	0.91	1.16	0.70	0.79	1.09	0.90	0.81	0.69	0.63	0.43	0.38	0.81	0.79
1.33	1.19	1.26	0.92	0.90	1.10	1.33	0.92	0.73	1.02	0.93	0.77	0.59	0.48
1.74	1.39	1.06	1.12	0.82	0.58	0.61	0.56	0.75	0.90	0.74	0.48	0.30	0.33

d Assay_II

IT-101_ sample 1

1.00		0.56		1.08	1.39	1.37	1.32	1.55	1.51	1.50	1.91
1.67	1.43	1.6	1.18	1.43	1.72	1.94	1.87	2.1	1.85	2.15	1.26
0.42	1.42	1.66	1.4	0.95	1.26	1.11	0.99	0.95	1.12	1.15	1.70
1.10	1.02	0.77	0.52	0.50	0.47	0.51	0.65	0.72	0.66	0.70	1.30

IT-101_ sample 2

1.00		0.51		1.08	1.39	1.37	1.32	1.55	1.51	1.50	1.91
0.32	1.48	0.96	0.99	0.74	0.55	0.70	0.90	1.22	1.08	0.86	1.07
0.51	1.64	1.07	0.59	0.80	0.86	0.97	0.94	0.87	0.64	0.71	1.25
0.82	0.66	0.72	0.75	0.61	0.67	0.78	0.83	0.69	0.64	0.65	1.15

Supplemental Fig. S4

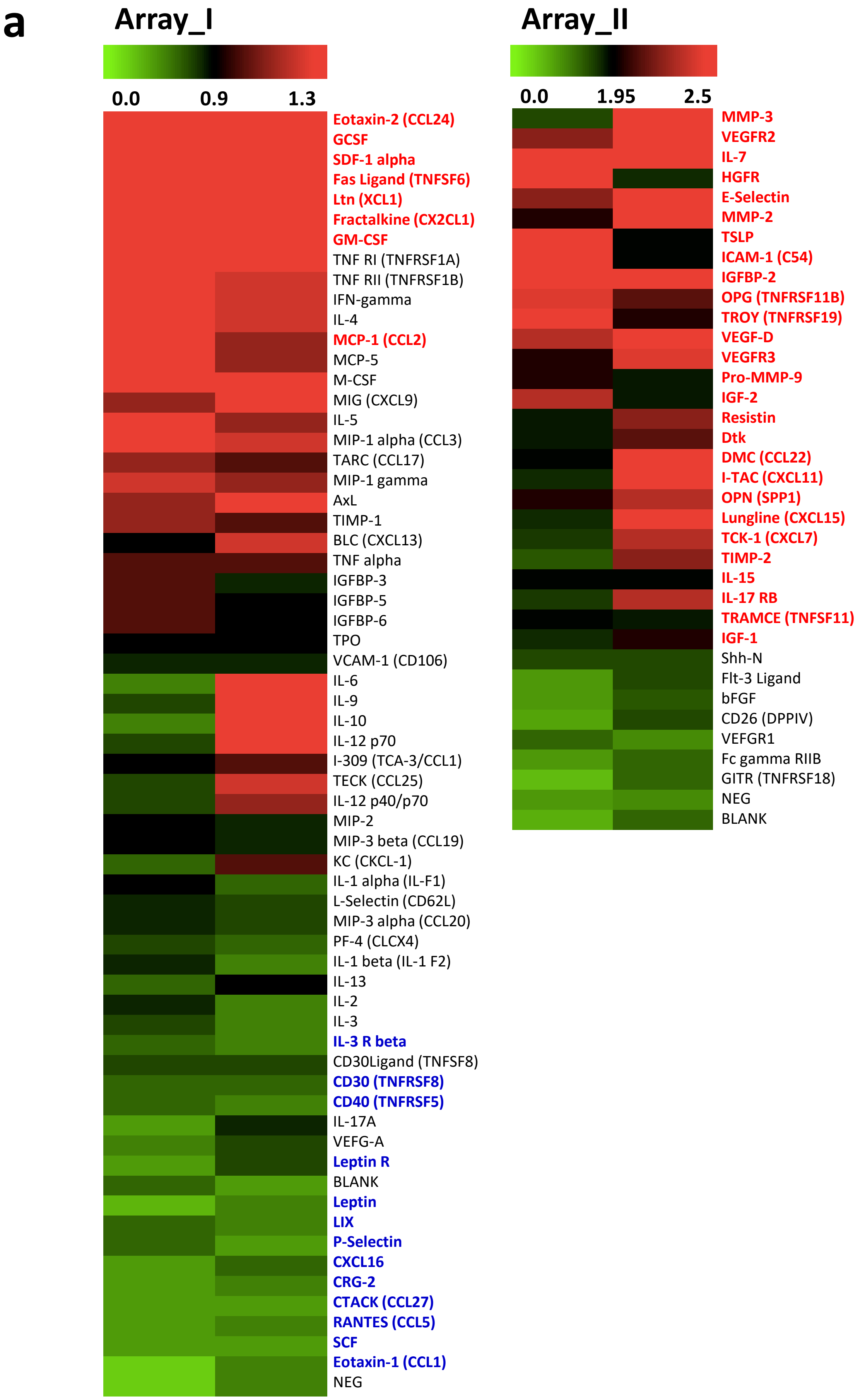

Supplemental Fig. S4_Continued

b Assay_I

POS		NEG		BLANK	AxL 1.14	BLC (CXCL13) 1.04	CD30 Ligand (TNFSF8) 0.69	CD30 (TNFRSF8) 0.59#	CD40 (TNFRSF5) 0.55#	CRG-2 (CXCL10) 0.47#	CTACK (CCL27) 0.40	CXCL16 0.53#	Eotaxin-1 (CCL11) 0.34#
Eotaxin-2 (CCL24) 3.14	Fas Ligand (TNFSF6) 2.19	Fractalkine (CX2CL1) 1.94	GCSF 1.99	GM-CSF 1.83	IFN-gamma 1.33	IGFBP-3 0.89	IGFBP-5 0.91	IGFBP-6 0.98	IL-1 alpha (IL-F1) 0.73	IL-1 beta (IL-1 F2) 0.63	IL-2 0.63	IL-3 0.63	IL-3 R beta 0.53
IL-4 1.36	IL-5 1.41	IL-6 1.13	IL-9 1.02	IL-10 0.91	IL-12 p40/p70 0.99	IL-12 p70 0.89	IL-13 0.72	IL-17A 0.62	KC (CKCL-1) 0.78	Leptin R 0.54	Leptin 0.36*	LIX 0.54	L-Selectin (CD62L) 0.74
Ltn (XCL1) 1.77	MCP-1 (CCL2) 1.54	MCP-5 1.33	M-CSF 1.19	MIG (CXCL9) 1.33	MIP-1 alpha (CCL3) 1.26	MIP-1 gamma 1.05	MIP-2 0.85	MIP-3 beta (CCL19) 0.83	MIP-3 alpha (CCL20) 0.74	PF-4 (CLCX4) 0.62	P-Selectin 0.49	RANTES (CCL5) 0.46*	SCF 0.40*
SDF-1 alpha 2.25*	TARC (CCL17) 1.24	I-309 (TCA-3/CCL1) 0.93	TECK (CCL25) 0.98	TIMP-1 1.23	TNF alpha 1.09	TNF RI (TNFRSF1A) 1.43	TNF RII (TNFRSF1B) 1.47	TPO 0.87	VCAM-1 (CD106) 0.80	VEFG-A 0.61	BLANK	BLANK	POS

c Assay_II

POS		NEG		BLANK	bFGF 1.13	CD26 (DPPIV) 1.13	Dtk 1.95	E-Selectin 2.51	Fc gamma RIIB 1.10	Flt-3 Ligand 1.21	GITR (TNFRSF18) 0.95
HGFR 2.41	ICAM-1 (C54) 2.37	IGFBP-2 2.68	IGF-1 1.86	IGF-2 2.04	IL-15 1.94	IL-17 RB 1.98	IL-7 2.71	I-TAC (CXCL11) 2.39*	Lungline (CXCL15) 2.07	DMC (CCL22) 2.53	MMP-2 2.46
MMP-3 3.45#	OPN (SPP1) 2.15*	OPG (TNFRSF11B) 2.26	Pro-MMP-9 1.87	Resistin 1.98	Shh-N 1.46	TCK-1 (CXCL7) 1.97	TIMP-2 1.82	TRAMCE (TNFSF11) 1.84	TROY (TNFRSF19) 2.25	TSLP 2.42	VEFGR1 1.15
VEGFR2 2.43	VEGFR3 2.17	VEGF-D 2.46	BLANK	BLANK	BLANK	BLANK	BLANK	BLANK	BLANK	BLANK	POS

d Assay_I

DMSO_sample 1　DMSO_sample 2　CPT_sample 1　CPT_ sample 2

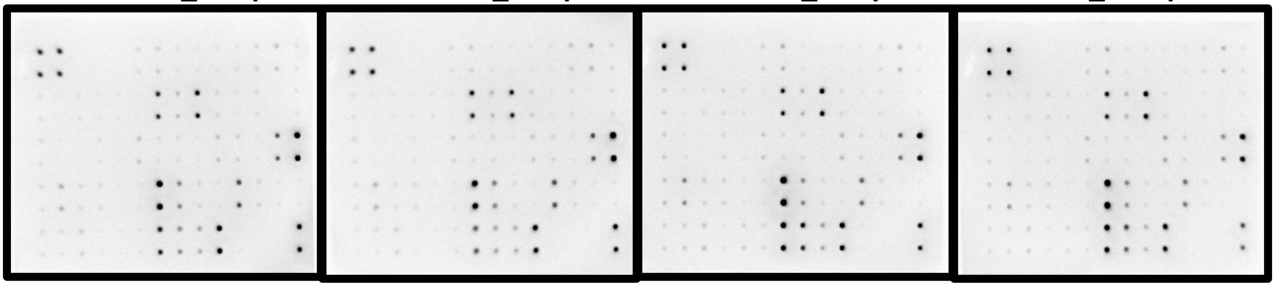

e Assay_II

DMSO_sample 1　DMSO_sample 2　CPT_sample 1　CPT_ sample 2

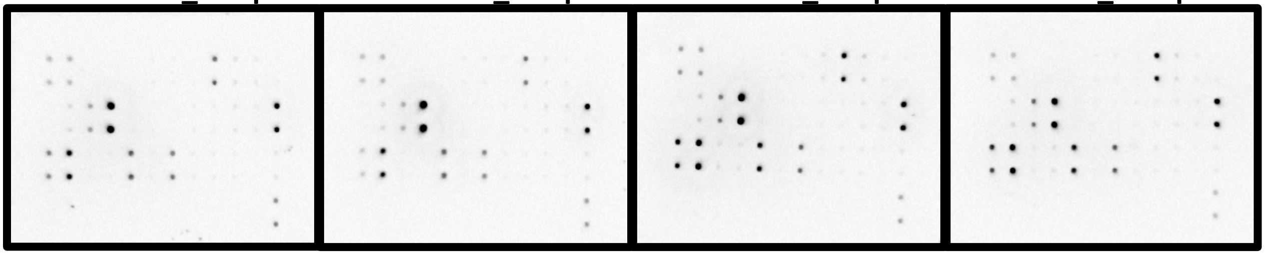

Supplemental Fig. S4_Continued

f Assay_I

CPT_sample 1

1.00		1.50		1.67	1.18	1.08	0.70	0.60	0.57	0.40	0.36	0.44	0.16
3.58	2.20	1.96	2.29	2.07	1.50	0.96	0.97	1.05	0.86	0.78	0.76	0.74	0.56
1.50	1.14	0.54	0.68	0.52	0.66	0.71	0.57	0.42	0.55	0.41	0.26	0.57	0.76
2.17	1.88	1.59	1.29	1.38	1.38	1.12	0.92	0.88	0.80	0.68	0.57	0.45	0.41
2.32	1.28	0.92	0.73	1.06	0.95	1.45	1.60	0.88	0.83	0.50	0.30	0.24	0.60

CPT_ sample 2

1.00		1.46		1.61	1.09	1.01	0.68	0.59	0.54	0.54	0.45	0.62	0.52
2.69	2.18	1.93	1.70	1.59	1.16	0.83	0.85	0.91	0.60	0.48	0.50	0.53	0.49
1.22	1.68	1.71	1.36	1.30	1.33	1.08	0.86	0.82	1.00	0.66	0.45	0.51	0.73
1.37	1.21	1.07	1.10	1.28	1.15	0.98	0.79	0.79	0.68	0.56	0.41	0.48	0.40
2.17	1.20	0.95	1.24	1.40	1.23	1.41	1.35	0.87	0.78	0.72	0.60	0.46	0.43

g Assay_II

CPT_ sample 1

1.00		0.86		0.66	0.88	0.79	1.82	2.16	0.91	0.93	0.58
3.09	2.81	2.62	1.74	2.30	1.94	1.63	2.16	1.69	1.67	1.92	1.98
3.61	1.97	2.38	1.97	1.81	1.47	1.64	1.42	1.87	2.48	2.91	1.33
1.49	1.96	2.34	2.96	2.11	2.31	1.70	1.49	1.26	1.24	1.44	3.22

CPT_ sample 2

1.00		0.98		1.29	1.37	1.47	2.08	2.87	1.30	1.48	1.33
1.73	1.93	2.74	1.97	1.77	1.94	2.34	3.26	3.09	2.46	3.13	2.94
3.30	2.33	2.14	1.77	2.15	1.46	2.31	2.23	1.81	2.01	1.93	0.98
3.36	2.37	2.58	3.49	3.57	4.41	4.99	4.28	3.97	5.13	5.57	2.56

Supplemental Fig. S4_Continued

Supplemental Fig. S4. Changes of cytokine expression after CPT treatment. (a) Using heat map to show the increase or decrease of cytokine levels after CPT treatment. **(b)(c)** Expression levels of cytokines and other secreted factors in mice with CPT treatment comparing with control group (treated with DMSO). Red indicates the value, measured with calculated control, which was more than 1.5, means significant increase after IT-101 treatment. Blue indicates the value, measured with calculated control, which was less than 0.6, means significant decrease after IT-101 treatment. "∗" indicates that the expression of factors in mice treated with IT-101 have similar trend to the expression of factors in mice treated with CPT. "#" indicates that the expression of factors in mice treated with IT-101 have opposite trend to the expression of factors in mice treated with CPT. **(d)** Probed membrane for RayBio C-Series Mouse Cytokine Antibody Array C3. **(e)** Probed membrane for RayBio C-Series Mouse Cytokine Antibody Array C4. **(f)** Semi-quantitative detection of 62 mouse proteins in serum using for RayBio C-Series Mouse Cytokine Antibody Array C3. **(g)** Semi-quantitative detection of 34 mouse proteins in serum using for RayBio C-Series Mouse Cytokine Antibody Array C4.

Supplemental Fig. S5

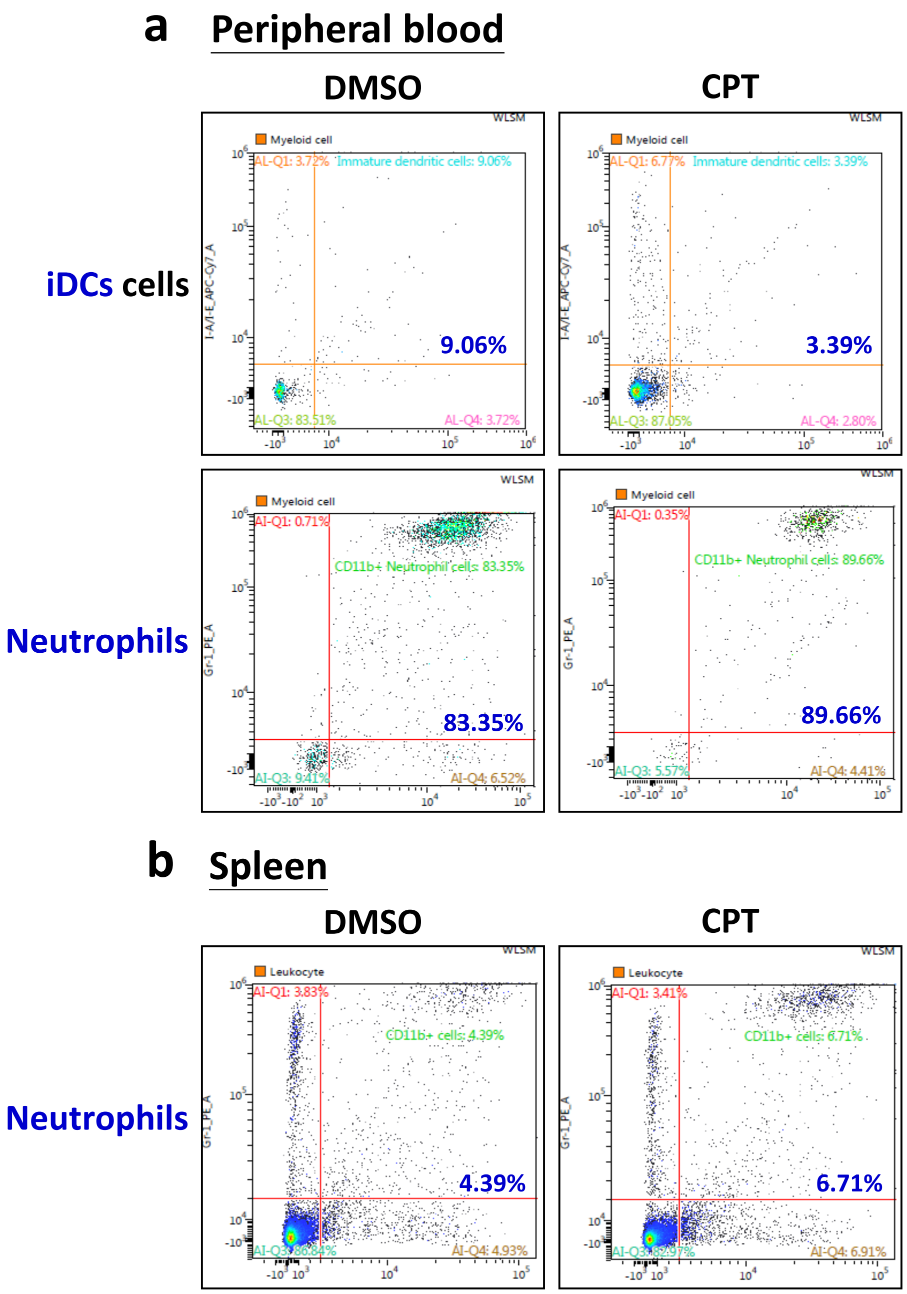

Supplemental Fig. S5. Significant changes after 216h treatment were presented by dot plots. Changes of cells populations in peripheral immune system after CPT (a) (b) and IT-101 (c)-(e) treatment. **(a)** In peripheral blood, the percentage of iDCs was decreased and the percentage of neutrophils was increased. **(b)** In spleen, the percentage of neutrophils was increased. **(c)** In lymph node, the percentage of iDCs was increased. **(d)** In peripheral blood, the percentage of B cells was decreased and the percentage of NK cells was increased. **(e)** In spleen, the percentage of NK cells and Tc cells was increased, and the percentage of Th cells was decreased.

Supplemental Fig. S5 _Continued

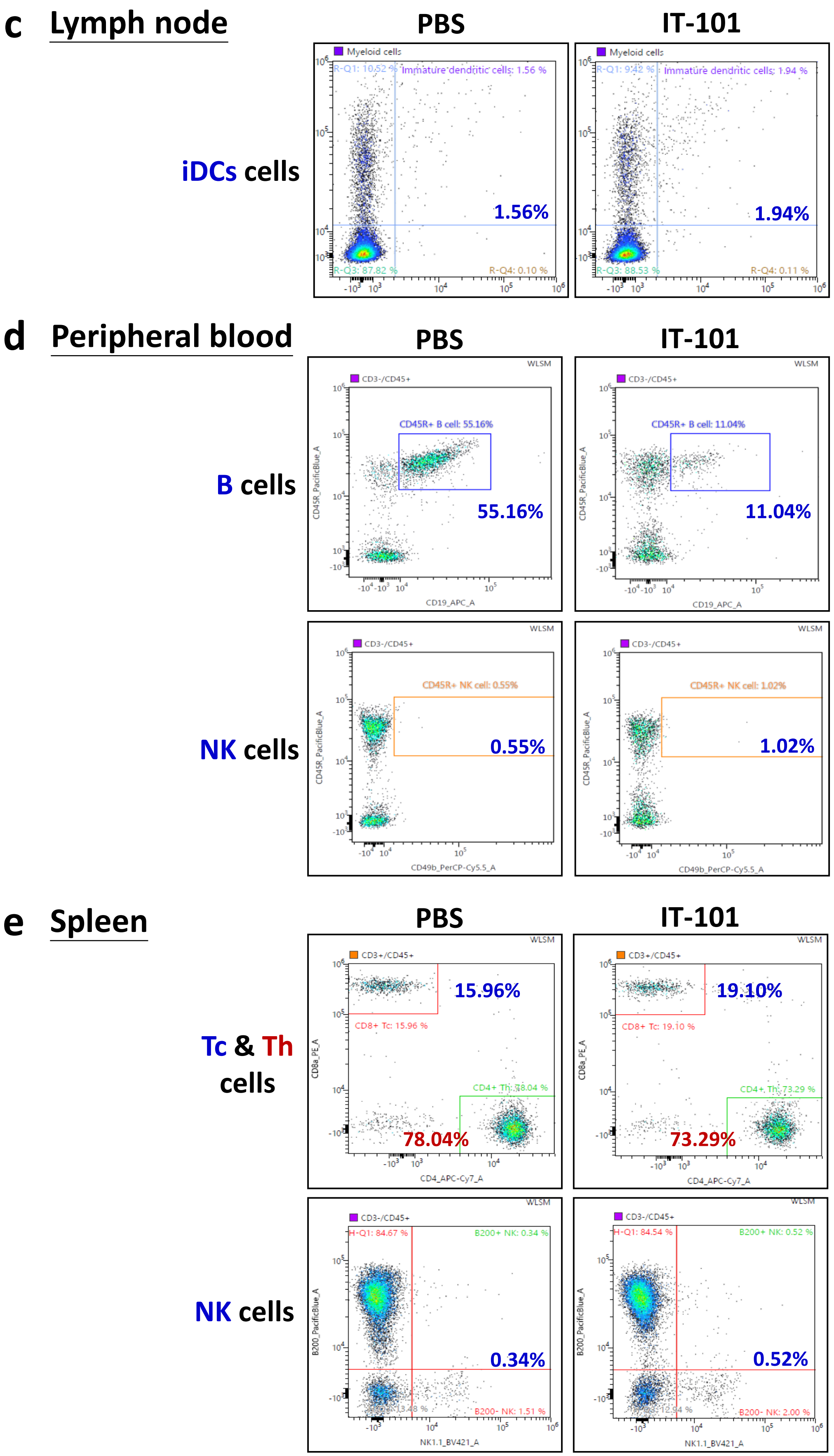

Supplemental Fig. S6

#1 **#2** **#3**

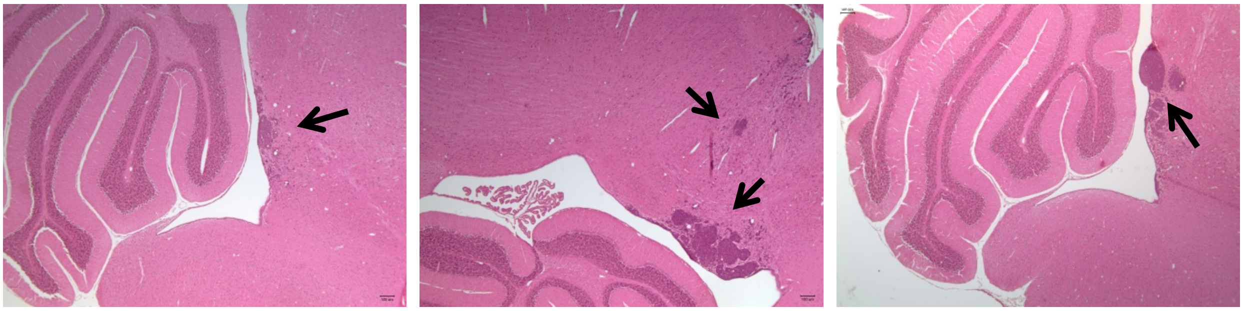

Supplemental Fig. S6. Early stage of tumorigenesis in F1B transgenic mice. The tumor was observed in brain of F1B transgenic mice at 2 months of age. #1, #2 and #3 indicate the images obtained from three mice.
